# The Efficacy of Injectable Atelocollagen in Arthroscopic Rotator Cuff Repair: A Systematic Review and Meta-Analysis

**DOI:** 10.7759/cureus.99255

**Published:** 2025-12-15

**Authors:** Mohamed Zahed, Ziad El Menawy, Mahmoud Elmesalmi, Nour Elnaggar, Farouk Ahmed, Mahmoud Odeh, Rawad M Azaz, Seifeldin H Amer, Mahmoud M Mourad, Ahmad Ali

**Affiliations:** 1 Orthopedics, John Radcliffe Hospital, Oxford University Hospitals NHS Trust, Oxford, GBR; 2 Trauma and Orthopedics, University Hospital of Wales, Cardiff, GBR; 3 Trauma and Orthopedics, St George's University Hospitals, London, GBR; 4 Medicine, Zagazig University, Zagazig, EGY; 5 Emergency Medicine, Queen Alexandra Hospital, Portsmouth, GBR; 6 Trauma and Orthopedics, Cardiff and Vale University Health Board, Cardiff, GBR; 7 Trauma and Orthopedics, London Royal free NHS trust, London, GBR; 8 General Practice, October 6 University, Giza, EGY; 9 Orthopedic Surgery, Minia university, Minia, EGY

**Keywords:** arthroscopic repair, atelocollagen, rotator cuff tear, systematic review and meta analysis, tendon healing

## Abstract

A rotator cuff (RC) tear can result in shoulder dysfunction and pain. Arthroscopic repair has been used for complete or advanced partial tears. However, the rates of retear and non-healing remain a challenge postoperatively. We aim to investigate the efficacy of injecting atelocollagen in combination with arthroscopic repair in patients with complete or partial RC tears.

We performed an electronic search using PubMed, Scopus, Web of Science, and the Cochrane Library up to the 7th of October 2025. Randomized controlled trials (RCTs) and cohort studies that evaluate atelocollagen as an adjunct to arthroscopic repair of RC tears were included. Primary outcomes were retear rate (Sugaya IV-V) and pain (visual analog scale (VAS)), and functional outcomes (American Shoulder and Elbow Surgeons score (ASES) and Korean Shoulder Score (KSS)) at 12 months. Additionally, we evaluated the overall risk of bias (ROB) of each included study using the RoB2 tool for RCTs and the ROBINS-I tool for cohorts. Pooled analyses were performed using Review Manager 5.4 with random-effects modeling applied when heterogeneity was detected.

Five studies, including a total of 743 patients, were included, with 376 using the intervention. We found a lower retear rate in the atelocollagen group that was statistically insignificant (RR=0.75, p=0.25). Additionally, the pooled analysis of VAS pain score showed a non-significant reduction in favor of atelocollagen (MD=-0.31, p=0.45). Functional outcomes at 12 months revealed no significant differences: ASES score (MD=-2.16, p=0.06) and KSS score (MD=0.26, p=0.91). For shoulder ROM, we detected non-significant differences in forward flexion (MD=-1.03°, p=0.73), external rotation (MD=-0.86°, p=0.74), or internal rotation (MD=0.06, p=0.81).

Despite statistical insignificance, atelocollagen administration during RC arthroscopic repair showed a consistent trend toward improved healing, particularly in retear rate and VAS pain. Further large and well-designed trials are warranted to confirm its clinical benefits.

## Introduction and background

Rotator cuff (RC) tear is a common orthopedic condition, affecting up to one-fifth of the general population [1]. It can cause shoulder pain and impair its range of motion (ROM), manifesting more often in the elderly [2]. Arthroscopic repair has been used as the gold standard surgery for the management of RC tears [3]. However, the retear rate and non-healing of the tendon-bone interface remain a challenge postoperatively [4].

Various biologics and scaffold materials were explored alone or in combination with surgery to provide a biomechanical and biochemical regenerative environment, including platelet-derived growth factors, stem cells, and atelocollagen [5,6]. Collagen is a fundamental structural protein in mammalian connective tissues, forming a major component of the skin, bones, tendons, ligaments, and cartilage [7]. It is made from a triple helix of three polypeptide chains, called tropocollagen [8]. Atelocollagen is produced by enzymatic removal of the N- and C-terminal telopeptides from type-1 collagen, which are responsible for the component's immunogenicity [9]. Therefore, it is a good candidate as a biological scaffold for RC tear repair, with potential to enhance cell adhesion, proliferation, and extracellular matrix formation [10].

Atelocollagen can be produced in several forms, including injectable gels, sponges, and membrane scaffolds [11]. Studies have investigated the injection of atelocollagen into complete or partial RC tears, either alone or in combination with arthroscopic repair [12]. Kim et al. (2020) investigated atelocollagen at 0.5ml and 1ml doses as a conservative management for 94 patients with partial RC tear [13]. By magnetic resonance imaging (MRI), they found a decrease in tear size at six months in favor of atelocollagen groups. Moreover, they found a statistically significant improvement in the Constant Shoulder Score (CSS), the American Shoulder and Elbow Surgeons score (ASES), and the visual analog scale (VAS) pain score in the intervention groups at the final visit.

Furthermore, the use of atelocollagen in combination with arthroscopic repair has been explored by Aldhafian et al., who included 129 patients with a complete RC tear and compared arthroscopic repair alone with arthroscopic repair combined with atelocollagen or acellular dermal matrix (ADM) injection [14]. They observed improvements in the three groups at 12 months in VAS, ASES, and CSS. However, the difference among them was not statistically significant.

These conflicting findings regarding the beneficial effects of atelocollagen, alone or in combination with arthroscopic repair, in RC tears prompted us to conduct this meta-analysis. We aim to pool studies that include patients with partial or full RC tears undergoing arthroscopic repair with atelocollagen injection vs. surgery alone, and to evaluate their clinical and radiological outcomes.

## Review

Methods

We performed this study following the methodologies outlined in the Cochrane Handbook of Systematic Reviews of Interventions [15]. Additionally, the Preferred Reporting Items for Systematic Reviews and Meta-Analyses (PRISMA) guidelines were meticulously followed for a standardized reporting [16].

Literature Search

Our systematic search was done across various electronic databases, including PubMed, Scopus, Web of Science (WOS), and the Cochrane Library, up to 7 Oct 2025. Our search strategy consisted mainly of the following keywords: (rotator cuff OR rotator cuff tear OR rotator cuff repair OR rotator cuff injury OR supraspinatus tear OR infraspinatus tear OR subscapularis tear OR teres minor tear OR shoulder tendon tear) AND (collagen OR atelocollagen OR collagen augmentation OR collagen scaffold OR collagen implant OR collagen injection OR biologic augmentation).

Inclusion Criteria

We included randomized controlled trials (RCTs) and prospective and retrospective cohort studies that enrolled patients diagnosed with partial- or full-thickness RC tears by preoperative imaging or intraoperative arthroscopy. Moreover, studies must have used injectable or gel-type atelocollagen as an intervention in combination with arthroscopic rotator cuff repair. On the other hand, the control group was patients undergoing arthroscopic repair alone and on conservative management.

Exclusion Criteria

Studies that used patch-type or sheet-type atelocollagen were excluded. Additionally, we excluded studies that did not perform arthroscopy. Moreover, we extended our exclusion criteria to include non-human or in vitro studies, only abstracts, cross-sectional designs, case reports, case series, non-English studies, and inaccessible full-text articles.

Screening and Study Selection

The articles retrieved from the database search were uploaded to the Rayyan software, where we removed duplicates and screened titles and abstracts to identify relevant articles. Secondly, we conducted a full-text screening of the initially included articles to ensure they met our eligibility criteria. Citation analysis was performed last to identify additional relevant publications. Two authors were individually responsible for the screening process, with a third serving as a tiebreaker in the event of a conflict.

Quality Assessment

We evaluated the risk of bias (ROB) using the appropriate tool for each study design. We used the Revised Cochrane Risk of Bias (RoB 2) tool for RCTs, which assesses five key domains: randomization process, deviations from the protocol, missing data, outcome measurement, and results selection [17]. Additionally, cohort studies were assessed using the Risk Of Bias In Non-randomized Studies - of Interventions (ROBINS-I) tool, which evaluates seven aspects related to confounding, participant selection, intervention selection and deviation, missing data, measurement of outcomes, and selection of the reported result [18]. Each domain was assessed according to the respective criteria of each tool to assign an overall judgment.

Data Extraction

Our summary sheet was based on extracting key characteristics from each study, including: study identification, country, study design, sample size, type of tear, form of atelocollagen used, comparator, follow-up duration, and imaging modality. Additionally, we collected patients' demographics, including mean age, sex distribution, tear size (anterior-posterior × medial-lateral, cm), Goutallier grade of fatty infiltration for each rotator cuff muscle, affected shoulder side, symptom duration, and surgical technique. Furthermore, the primary outcomes extracted were retear rate at final follow-up, defined radiologically as Sugaya classification types IV-V, and clinical outcomes, including pain intensity measured by the VAS at the endpoint and shoulder function at 12 months, assessed using the ASES score and the Korean Shoulder Score (KSS). Secondary outcomes included shoulder ROM parameters at 12 months, including forward flexion (or elevation), external rotation at the side, and internal rotation (measured as the highest vertebral level reached behind the back).

Statistical Analysis

Our analysis was conducted using the Review Manager software (RevMan 5.4). Categorical data were reported as risk ratios (RRs) and 95% confidence intervals (CIs), whereas continuous outcomes were reported as mean differences (MDs) and 95% CIs. Additionally, we assessed statistical heterogeneity using the I-squared (I2) and Chi-squared (Chi2) statistics, a forest plot, and a random-effects model for heterogeneous outcomes. Furthermore, we performed a subgroup analysis by tear type. 

Results

Literature Search

**Figure 1:**
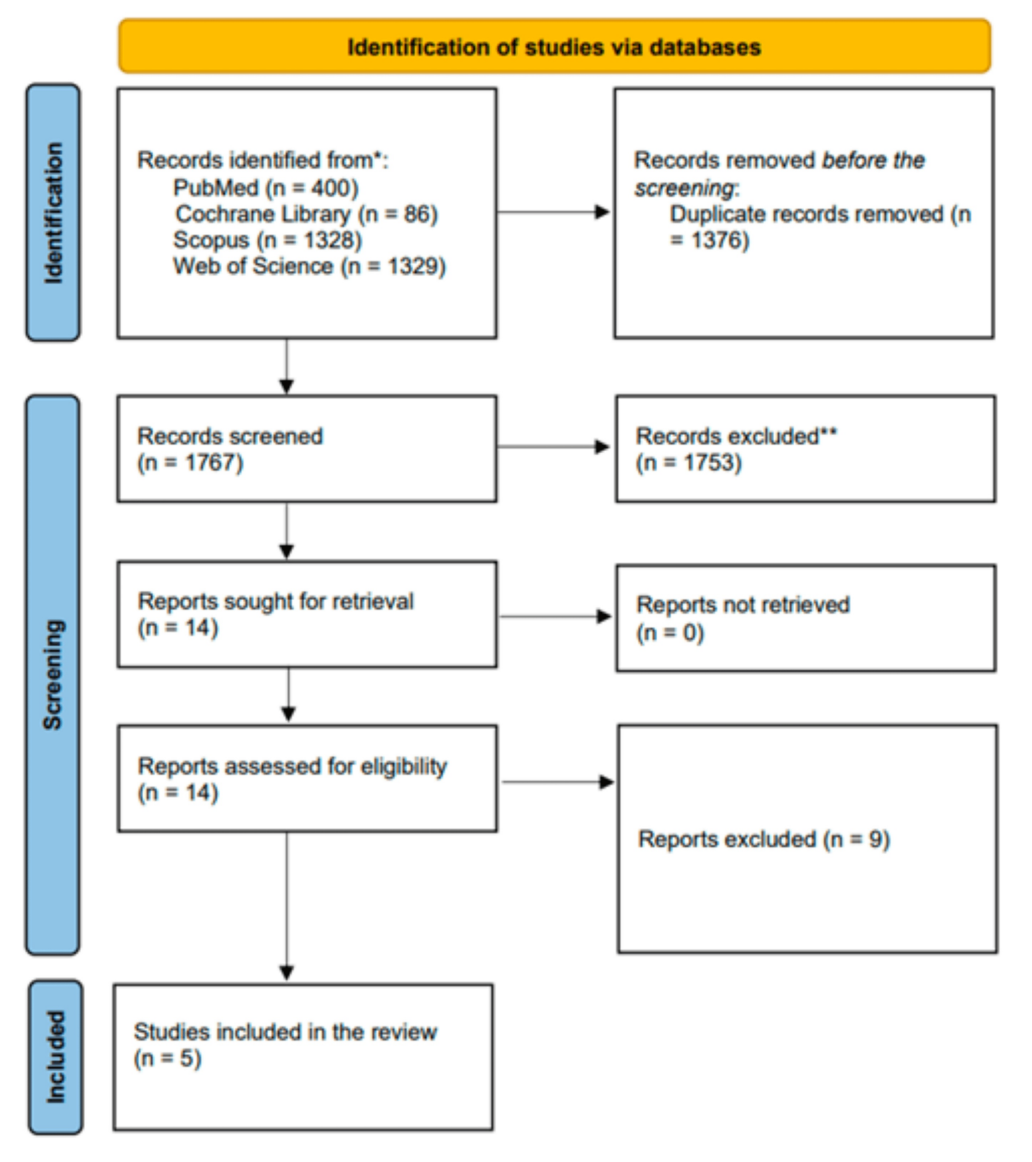
PRISMA flow diagram detailing the screening process of the included studies References: [14,19–22] PRISMA - Preferred Reporting Items for Systematic Reviews and Meta-Analyses

We identified 3143 articles during the initial screening, of which we removed 1376 duplicates. The remaining articles underwent title and abstract screening, yielding 14 articles that met our predetermined criteria. Consequently, we retrieved the full texts of these articles, resulting in five included studies for the meta-analysis [14,19-22]. More details on the literature search and selection process are shown in Figure 1.

Characteristics of the Included Studies and Baseline Demographics

The five included studies were conducted in South Korea between 2019 and 2024, including one RCT and four retrospective cohort studies (Table 1). Across all studies, patients with partial- or full-thickness RC tears were treated with arthroscopic repair and intraoperative injection of gel-type I atelocollagen at the tendon-bone interface, in volumes ranging from 1 to 3 mL. In contrast, the control group did not receive collagen augmentation. All patients were adults, with a mean age of 58 to 63 years. Most studies report small- to medium-sized full-thickness supraspinatus tears in the right shoulder, with follow-up durations of 12 to 27 months. Moreover, patients' tears were within the range of repairable cuff pathology, with a mean tear length of 1-2 cm in the anteroposterior direction and 1-2.5 cm in the mediolateral direction. Additionally, minimal to moderate muscle atrophy was evident, with grades 0-2 predominating in the Goutallier classification (Table 2). 

**Table 1:** Summary of the characteristics of included studies References: [14,19–22] RCT - randomized controlled trial; ADM - acellular dermal matrix; MRI - magnetic resonance imaging; HA - hyaluronic acid; PASTA - partial articular supraspinatus tendon avulsion; A-P - anterior-posterior; M–L - medial-lateral; SD - standard deviation; I - intervention; C - control; T - tesla (MRI strength)

Study (author, year)	Country	Study design	Sample size (intervention/ control)	Tear type	Atelocollagen form	Comparator	Follow-up duration (months, mean ± SD)	Imaging modality
Aldhafian et al., 2023 [14]	South Korea	Retrospective cohort	Group 1: 36 (arthroscopic repair only) Group 2: 44 (Atelocollagen) Group 3: 49 (ADM Allograft)	Full-thickness supraspinatus tears (<5 cm), repairable	Injectable/Gel-Type Type I Atelocollagen, 1 mL intraoperative injection at tendon-bone interface	Standard arthroscopic repair without augmentation	I: 20.0 ± 6.3, C: 21.6 ± 5.1	MRI (2 and 12 months post-op)
Jeong et al., 2021 [19]	South Korea	Prospective RCT	22 (Atelocollagen + HA) / 22 (Control, No Injection)	Full-thickness rotator cuff tears (repairable, type I)	Injectable Type I atelocollagen (3 mL, co-administered with 3 mL HA)	Standard arthroscopic repair without atelocollagen or HA	12.1 ± 1.5 (I), 11.8 ± 0.8 (C)	MRI at 12 months (3.0T, Sugaya classification)
Ji et al., 2023 [20]	South Korea	Retrospective cohort	68 / 68	High-grade partial articular supraspinatus tendon avulsion (PASTA) lesions (Ellman grade 3)	Injectable / Gel-type porcine Type I Atelocollagen (1 mL, 3 mg/mL)	Transtendon repair without augmentation	≥ 24 months (minimum 2 years)	MRI (6 months, 1 year)
Kim et al., 2019 [22]	South Korea	Retrospective cohort	61 / 60	Full-thickness supraspinatus tear (± infraspinatus/subscapularis)	Injectable/Gel-type Type I porcine atelocollagen (RegenSeal, 3 mL) applied intraoperatively at bone-tendon interface	Standard arthroscopic rotator cuff repair without atelocollagen	26.7 months (mean), MRI at 6.4 months	MRI (1.5 T) using the Sugaya classification for patients.
Kim et al., 2024 [21]	South Korea	Retrospective propensity score-matched comparative study	181 / 181	Small- to medium-sized full-thickness rotator cuff tears (DeOrio&Cofield classification)	Injectable atelocollagen (3 mL, Coltrix Tendoregen, Ubiosis) injected at bone-tendon interface	Arthroscopic rotator cuff repair without atelocollagen injection	22.5 ± 9.0 (I) / 21.8 ± 9.6 (C)	MRI (3.0-T, postoperative at 6 months)

**Table 2:** Baseline demographic and clinical characteristics of patients References: [14,19–22] A-P - anterior-posterior; M-L - medial-lateral; RCR - rotator cuff repair; MRI - magnetic resonance imaging; GH - glenohumeral; AC - acromioclavicular; OA - osteoarthritis; SLAP - superior labrum anterior-posterior; DR-SB - double-row suture bridge; PS - propensity score; SD - standard deviation; wk - week(s)

Study	Group	Mean age (years)	Male (%)	Tear size (cm, A–P × M–L)	Goutallier stages, grade 0:1:2:3:4			Affected shoulder (right:left)	Symptom duration (months)	Surgical technique
					Subscapularis	Supraspinatus	Infraspinatus			
Aldhafian et al., 2023 [14]	Intervention	63.0 ± 8.0	(20) 45.5 %	A–P = 1.9 ± 0.9 M–L = 2.1 ± 0.9	8:23:11:2:0	5:23:15:1:0	6:27:10:1:0	NA	Not reported	Predominantly double-row suture bridge (some single-row Mason-Allen); biceps tenotomy/tenodesis for associated biceps pathology as needed
	Control	63.2 ± 8.3	(10) 27.8 %	A–P = 1.5 ± 0.7 M–L = 2.0 ± 0.9	2:28:6:0:0	1:10:20:3:2	3:12:20:1:0	NA		
Jeong et al., 2021 [19]	Intervention	58.8 ± 5.4	(10) 45.5%	Not specified (complete repair, type I,<5 cm)	NA	NA	NA	17:05	Not reported	Arthroscopic double-row transosseous equivalent (type I repair covering full footprint)
	Control	58.4 ± 6.5	(12) 54.5%		NA	NA	NA	14:08		
Ji et al., 2023 [20]	Intervention	61.4 ± 9.2	(27) 39.7%	A–P = 1.8 ± 0.68, M–L = 1.2 ± 0.50	NA	NA	NA	36:32	≥6 months failed conservative treatment	Arthroscopic transtendon suture-bridge repair ± atelocollagen injection
	Control	61.9 ± 7.9	(29) 42.6%	A–P = 1.8 ± 0.80, M–L = 1.1 ± 0.49	NA	NA	NA	30:38		
Kim et al., 2019 [22]	Intervention	59.8 ± 7.3	(26) 42.6%	A–P = 1.93 ± 1.16, M–L = 2.46 ± 1.16	20:37:4:0:0	6:48:7:0:0	2:44:14:0:0	NA	20.5 ± 45.3 (I) / 9.5 ± 14.6 (C)	Arthroscopic suture-bridge repair with acromioplasty; injectable gel-type atelocollagen placed between bone and tendon
	Control	57.8 ± 8.1	(31) 51.7 %	A–P = 1.96 ± 1.19 M–L = 2.43 ±1.35	17:43:0:0:0	12:41:6:0:1	12:32:13:2:1	NA		
Kim et al., 2024 [21]	Intervention	59.8 ± 9.0	(92) 50.8%	A–P = 1.05 ± 0.56, M–L = 1.25 ± 0.72	119:54:5:1:2	NA	135:42:3:1	NA	≥3 months failed conservative treatment	Arthroscopic double-row suture bridge repair (DR-SB); 3 mL injectable atelocollagen at bone-tendon interface; biceps tenodesis/tenotomy as indicated

Quality of the Included Studies

**Figure 2:**
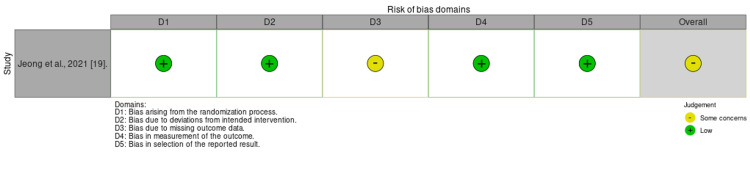
ROB summary for the RCT assessed using the RoB 2 tool Reference: [19] ROB - risk of bias; RCT - randomized controlled trial

**Figure 3:**
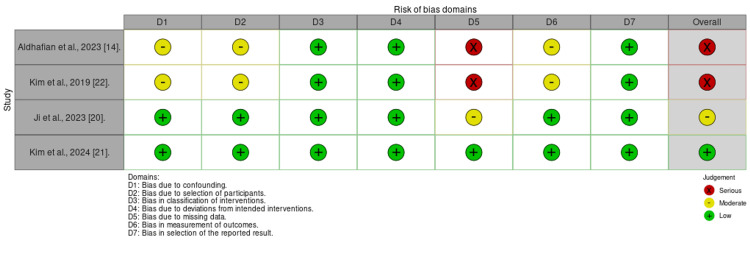
ROB assessment for cohort studies using the ROBINS-I tool References: [14,20–22] ROB - risk of bias; ROBINS-I - Risk Of Bias In Non-randomized Studies - of Interventions

According to the ROBINS-I tool, two studies were at serious risk, one at moderate risk, and one at low risk. Additionally, the fifth study was found to have some concerns under the ROB 2 criteria. Further demonstration is shown in Figures 2 and 3.

Clinical Outcomes

**Figure 4:**
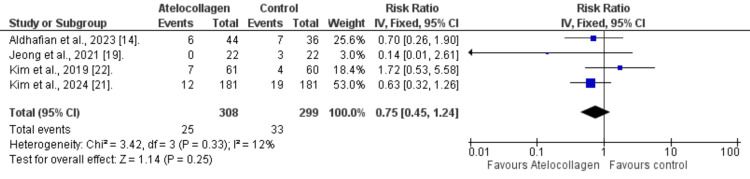
Retear rate References: [14,19,21,22]

Retear rate (radiologic outcome): Across four studies, the rate of retear in the atelocollagen group was lower than in the control group (25/308 vs. 33/299), having a 25% relative reduction. However, no statistically significant difference was found (RR=0.75 (95% CI: 0.45-1.24; p=0.25), Figure 4. Additionally, minimal heterogeneity was observed among the included studies (I²=12%).

**Figure 5:**
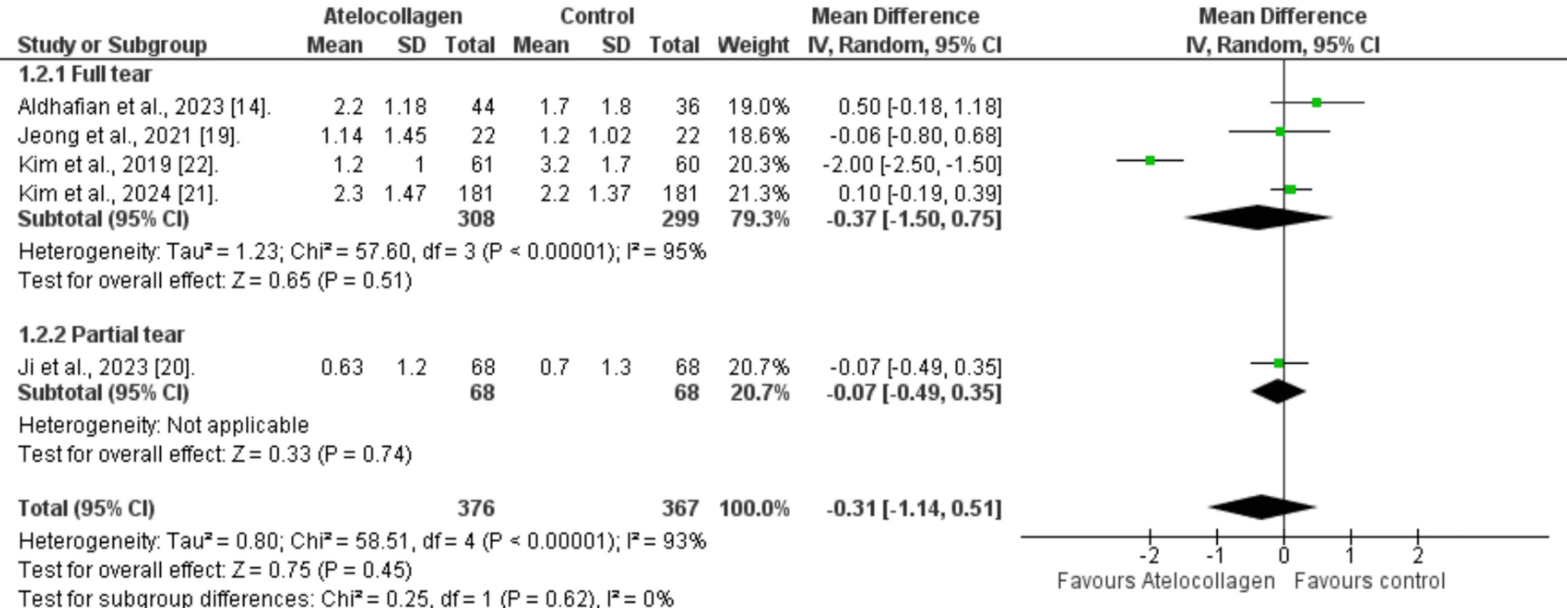
Forest plot comparing VAS pain score References: [14,19-22] VAS - visual analog scale

Pain assessment (VAS): The atelocollagen group showed a lower VAS score, but the difference was not statistically significant (MD=-0.31, 95% CI: -1.14 to 0.51, p=0.45; Figure 5). Additionally, marked heterogeneity was detected in the overall pooled results (I²=93%, p<0.00001). To resolve this heterogeneity, we subcategorized the VAS by the tear type. Patients with full-thickness tears also show a non-significant difference between groups (MD=-0.37, 95% CI: -1.50 to 0.75, p=0.51). Additionally, comparable results were found for the partial-thickness tear subgroup reported by Ji et al. (2023; MD=-0.07, 95% CI: -0.49 to 0.35, p=0.74).

Functional Scores at 12 Months

**Figure 6:**
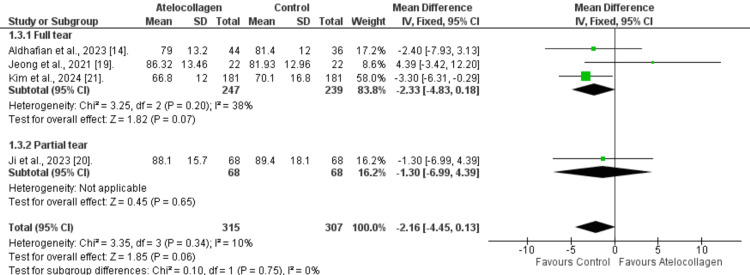
Twelve-month comparison of the ASES score forest plot References: [14,19-21] ASES - American Shoulder and Elbow Surgeons

ASES score​​​​​: The pooled analysis of four included studies revealed a non-significant difference in ASES score at 12 months between the atelocollagen and the control group (MD=-2.16, 95% CI: -4.45 to 0.13, p=0.06; Figure 6). Additionally, we found low heterogeneity among studies (I²=10%, p=0.34). Furthermore, full-thickness or partial-thickness tear subgroups were also statistically insignificant (MD=-2.33, 95% CI: -4.83 to 0.18, p=0.07, and MD=-1.30, 95% CI: -6.99 to 4.39, p=0.65, respectively).

**Figure 7:**
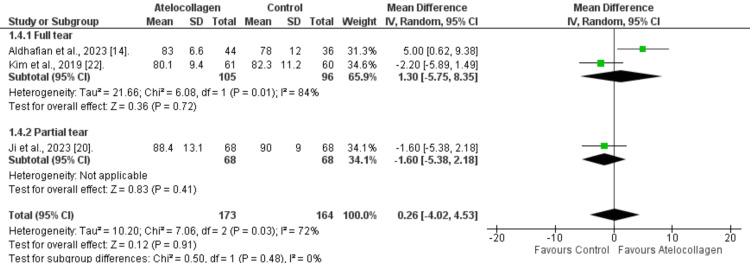
KSS score forest plot at 12 months References: [14,20,22] KSS - Korean Shoulder Score

KSS score: Across three studies, the KSS score was comparable between the atelocollagen and the control groups (MD=0.26, 95% CI: -4.02 to 4.53, p=0.91; Figure 7). High heterogeneity was noticed over the pooled result (I²=72%, p=0.03). Moreover, the full-thickness or partial-thickness tear subgroups show statistical insignificance (MD=1.30 (95% CI: -5.75 to 8.35, p=0.72), and MD =-1.60 (95% CI: -5.38 to 2.18, p=0.41), respectively).

Range of Motion (ROM) at 12 Months

**Figure 8:**
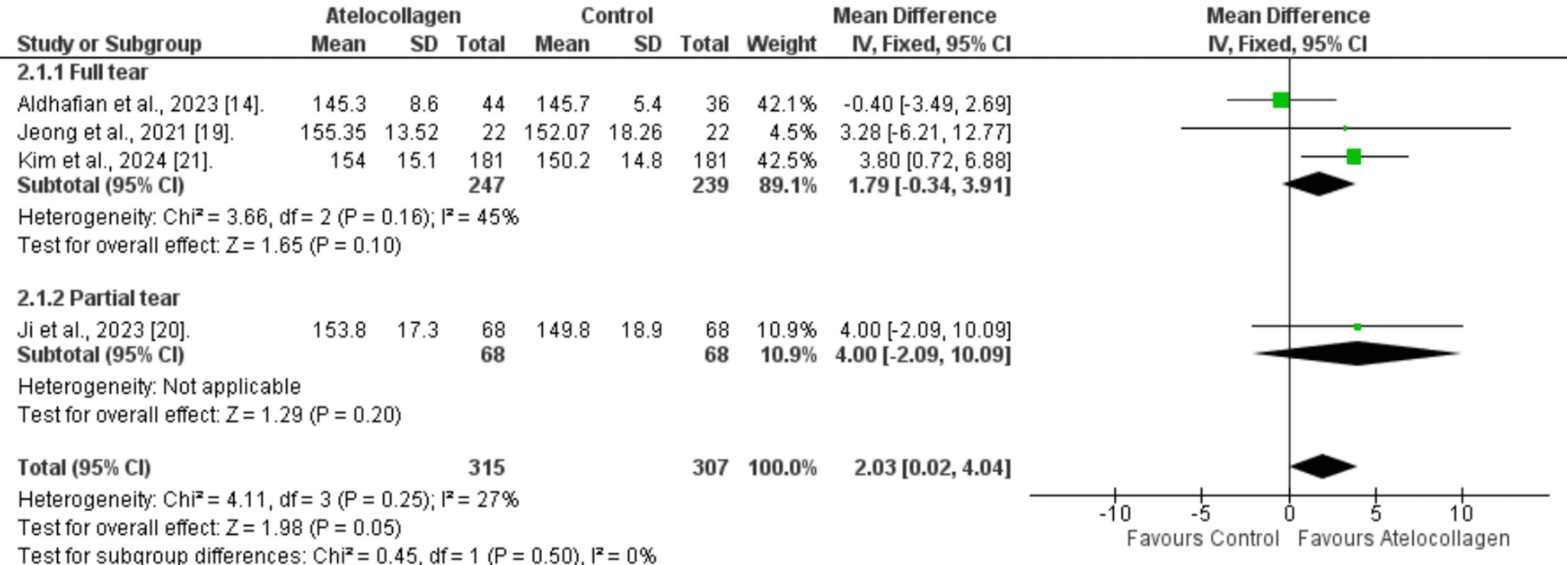
Forest plot of forward flexion comparison at 12 months References: [14,19-21]

Forward flexion:* *The atelocollagen group demonstrated a comparable improvement in flexion ROM (MD=-1.03°, 95% CI: -6.91 to 4.86°, p=0.73) (Figure 8). Also, significant heterogeneity was present (I²=82%, p=0.0007). Additionally, insignificance was noticed in the subgroups (MD=-2.62°, 95% CI: -9,39 to 4.14°, p=0.45 for full-thickness tear and MD=4.00°, 95% CI: -2.09 to 10.09°, p=0.20 for partial-thickness tear).

**Figure 9:**
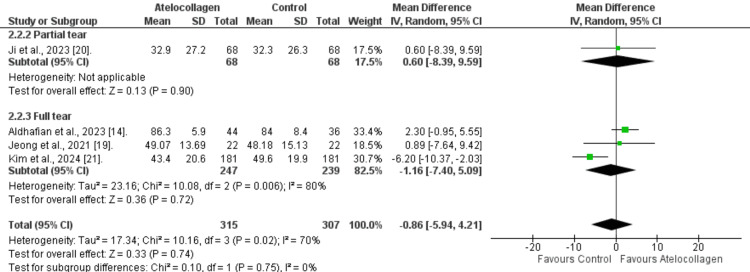
Forest plot of external rotation comparison at 12 months References: [14,19-21]

External rotation: Slightly greater external rotation was observed, favoring the control group (MD=-0.86°, 95% CI: -5.94 to 4.21°). However, we did not observe statistical significance (p=0.74) (Figure 9). Additionally, significant heterogeneity was found (I²=70%, p=0.02). Subgroup analysis was also insignificant (MD=-1.16°, 95% CI: -7.40 to 5.09°, p=0.72 and MD=0.60°, 95% CI: -8.39 to 9.59°, p=0.90) for full-thickness and partial tear, respectively.

**Figure 10:**
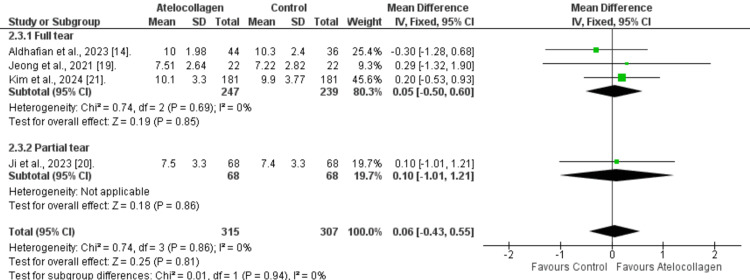
Forest plot of internal rotation comparison at 12 months References: [14,19-21]

Internal rotation: A slightly greater internal rotation was observed in favor of the atelocollagen group, despite being insignificant statistically (MD=0.06, 95% CI: -0.43 to 0.55, p=0.81) (Figure 10). Additionally, the overall pooled results showed very low heterogeneity (I²=0%, p=0.86). Subgroup analysis also revealed no significant differences for either full-thickness tears (MD=0.05, 95% CI: -0.50 to 0.60, p=0.85) or partial-thickness tears (MD=0.10, 95% CI: -1.01 to 1.21, p=0.86).

Discussion

In this study, we explored the potential benefits of atelocollagen augmentation in patients with repairable RC tears who are undergoing arthroscopic surgery. Across five included studies, we observed trends toward lower retear rates and higher VAS scores in favor of the atelocollagen group, although these differences did not reach statistical significance. This was also observed in the functional outcomes, including ASES and KSS scores at 12 months, which showed a potential benefit but were not statistically significant. Regarding shoulder mobility, we did not observe a substantial difference between the groups in forward flexion, external rotation, or internal rotation. Furthermore, subgroup analyses by tear type did not show an effect on the overall outcome, consistent with findings across different tears.

Although the pooled outcomes for retear rate, pain relief, and functional recovery were not statistically significant, the consistent trend of improvement with atelocollagen is clinically encouraging. The retear events, in particular, were found to be a major barrier to these operations, with retear risks of 20% and 40% even for small-to-medium tears [23]. Therefore, the observed trend toward reduced rates with atelocollagen (25% relative reduction) warrants continued investigation and larger trials to confirm, particularly given the homogeneity of the included studies on this outcome.

Additionally, the slightly inferior ROM results in the atelocollagen group can be explained by the transient postoperative stiffness resulting from atelocollagen's role in collagen deposition and remodeling at the tendon-bone interface [10]. Our findings, if longitudinally proved, could also suggest long-term durability despite the early restricted ROM.

Moreover, other qualities of atelocollagen support its potential therapeutic role. This is reflected in its excellent biocompatibility and minimal antigenicity, resulting from the removal of telopeptides and its purified type 1 collagen nature [10,24]. Moreover, we did not observe any adverse effects in the atelocollagen group across the included studies, reinforcing its potential as a long-term complement to conventional arthroscopic repair.

A critical aspect of our analysis was the distinction between full and partial-thickness tears. Ji et al., the only study with a partial tear population, reported slightly greater forward flexion and abduction at final follow-up (p<0.05), as well as increased tendon thickness on MRI at 6 and 12 months postoperatively [20]. However, the functional scores were comparable between the two groups, which suggests that atelocollagen can improve tendon healing quality, even when early functional outcomes appear equivalent. Moreover, the improvement in shoulder mobility could be explained by their inclusion of partial-thickness PASTA lesions, which may heal more readily and demonstrate early functional improvement, given the preservation of the native tendon.

Another key subgroup relied on Jeong et al., the only included RCT, which compared the combination of hyaluronic acid and atelocollagen to no injection [19]. They reported better results with the combination in terms of VAS and ASES scores, forward elevation, and external and internal rotation, although the differences were not statistically significant. Additionally, they did not find any retear events in the atelocollagen group, vs. 9.5% in the no-injection group. The discrepancy between these findings, our pooled analysis, and some of the included studies may have stemmed from the synergistic effect of hyaluronic acid, which could have aided in reducing inflammation and organizing the extracellular matrix during the healing process. Additionally, the study design, as the only RCT, may have reduced confounding and optimized the effect of standardized surgical and rehabilitation protocols on healing.

Other biologics were investigated to enhance tendon-to-bone healing in patients with RC tears. In this regard, Gill et al. recently conducted a meta-analysis of the combination of platelet-rich plasma (PRP) with arthroscopic repair across 22 studies, which matched our population and control [25]. They reported a significant reduction in pain at both 6 and 12 months (MD=0.34 (0.10-0.59) and MD=0.24 (0.03-0.44), respectively). This superiority in comparison with ours may have stemmed from the longer follow-ups, particularly given that our VAS was mainly measured shortly post-operatively. This was noted in their three-month VAS scores, which were not significant, as ours were (MD=-0.11 (-1.14, 0.93)). However, both biologics are thought to exert their pain relief through a shared mechanism. While PRP exerts its analgesic effects through the release of protease-activated receptor four peptides, atelocollagen is also believed to possess analgesic and anti-inflammatory properties similar to those of PRP [10,26]. Additionally, the fact that all the included studies injected atelocollagen at the tendon-bone interface intraoperatively, rather than in the subacromial space, may also have contributed to the pain reduction.

Similar to our findings, they did not find a significant difference between the PRP and control groups in ASES scores at 12 months. Moreover, the benefits of PRP were clearly evident in the reduction in retear rate observed at 25 months (MD=15.03). Compared with these findings, our pooled analysis of the retear rate suggests a trend toward favoring atelocollagen, although the difference is not statistically significant. However, the number of included studies and the limited statistical power in our analysis can explain this difference.

Acellular collagen matrix patch (ACMP) was also explored in conjunction with arthroscopy and found to outperform arthroscopy alone in retear rate and ASES, as reported in a meta-analysis of five RCTs (p<0.05) [27]. The mechanistic approach of each intervention can explain the difference between our study and theirs. While atelocollagen provides biochemical support, ACMP offers mechanical and structural support. Moreover, the majority of our studies were cohort studies, unlike the RCTs' inclusion criteria, which could have influenced the outcomes.

Another report by Warren et al. investigated the bioinductive patch across 13 clinical studies and found significant improvements in ASES and VAS pain scores, with final ASES scores increasing from approximately 45-50 preoperatively to 83-96 postoperatively and VAS decreasing from five to seven to around one [28]. Moreover, the overall retear rate was notably lower (8.3% for full-thickness and 1.1% for partial-thickness repairs) compared to standard arthroscopic repair. The results of this study could also be attributed to the nature of the bioinductive patch, which provides mechanical reinforcement and serves as a biological scaffold. At the same time, atelocollagen is a biochemical stimulus that enhances cellular healing without providing structural support or strength.

To our knowledge, this is the first systematic review and meta-analysis to investigate the efficacy of atelocollagen administration in arthroscopic RC tears. We included studies with comparable surgical techniques, follow-up durations, and outcome measures, thereby providing some homogeneity among the included studies. However, we acknowledge several limitations. First, the low sample size stemming from limited evidence may have reduced the statistical power. Therefore, it prevented the detection of subtle but clinically meaningful differences between the groups. Second, including only one RCT may have introduced selection and performance biases. Third, the differences in tear sizes may have decreased the direct comparability among studies. Fourth, all included studies were performed in South Korea, limiting our ability to generalize the findings to other populations and care settings. Finally, variations in dosage and form, as well as inconsistencies in rehabilitation protocols, may have impacted the net outcome.

## Conclusions

This systematic review and meta-analysis evaluated the efficacy of injectable atelocollagen as an adjunct to arthroscopic rotator cuff repair in patients with partial- or full-thickness tears. While our pooled analysis did not demonstrate statistically significant differences, consistent trends favoring atelocollagen were observed across retear rates, postoperative pain, and functional outcomes. The lack of statistical significance may be attributed to the limited available studies, small sample sizes, and heterogeneity in surgical protocols. Despite these limitations, the directional consistency of findings, combined with atelocollagen's biological properties supporting tendon-bone healing through enhanced cell adhesion and extracellular matrix formation, suggests potential clinical utility. Large-scale, multicenter randomized controlled trials with standardized protocols and longer follow-up periods are essential to definitively establish the clinical benefits of atelocollagen in rotator cuff repair and identify patients most likely to benefit from this intervention.
